# Reporter gene comparison demonstrates interference of complex body fluids with secreted luciferase activity

**DOI:** 10.1038/s41598-020-80451-6

**Published:** 2021-01-14

**Authors:** M. Neefjes, B. A. C. Housmans, G. G. H. van den Akker, L. W. van Rhijn, T. J. M. Welting, P. M. van der Kraan

**Affiliations:** 1 Experimental Rheumatology, Department of Rheumatology, Radboud University Medical Centre, Nijmegen, The Netherlands; 2 Laboratory for Experimental Orthopedics, Department of Orthopedic Surgery, Maastricht University, Maastricht, The Netherlands; 3 Laboratory for Experimental Orthopedics, Department of Orthopedic Surgery, Maastricht University Medical Centre+, P.O. Box 5800, 6202 AZ Maastricht, The Netherlands

**Keywords:** Assay systems, Cell signalling, High-throughput screening, Reporter genes

## Abstract

Reporter gene assays are widely used to study cellular signaling and transcriptional activity. Few studies describe the use of reporter genes for studying cellular responses on complex body fluids, such as urine and blood. Selection of the optimal reporter gene is crucial for study outcome. Here, we compared the characteristics of five reporter genes (Firefly luciferase, stable- and unstable Nano luciferase, secretable *Gaussia* luciferase and Red Fluorescent Protein) to study complex body fluids. For this comparison, the NFκB Response Element (NFκB-RE) and Smad Binding Element (SBE) were identically cloned into the five different reporter vectors. Reporter characteristics were evaluated by kinetic and concentration–response measurements in SW1353 and HeLa cell lines. Finally, reporter compatibility with complex body fluids (fetal calf serum, knee joint synovial fluid and human serum) and inter-donor variation were evaluated. Red Fluorescent Protein demonstrated poor inducibility as a reporter gene and slow kinetics compared to luciferases. Intracellularly measured luciferases, such as Firefly luciferase and Nano luciferase, revealed good compatibility with complex body fluids. Secreted *Gaussia* luciferase appeared to be incompatible with complex body fluids, due to variability in inter-donor signal interference. Unstable Nano luciferase demonstrated clear inducibility, high sensitivity and compatibility with complex body fluids and therefore can be recommended for cellular signaling studies using complex body fluids.

## Introduction

The development of reporter gene assays has greatly facilitated gene expression and cell signaling studies. By using transcription factor-binding response elements coupled to a reporter gene, activation of signaling pathways can be studied upon stimulation. However, measuring such responses induced by a complex body fluid can be challenging due to viscosity, ionic strength and the proteolytic milieu of the sample^1,2^. A regularly used body fluid is serum and this easily accessible fluid can be used for measurements related to many diseases, such as cancer and heart conditions^3,4^. Another typical example is synovial fluid, a viscous fluid found in the intra-articular space of synovial joints, and which is compositionally altered in patients with osteoarthritis^5^.

Different reporter genes are available and in the past mostly radioactive assays were used such as chloramphenicol acetyltransferase^6^. However, nowadays luciferases and fluorescent proteins are most commonly used as these are far less time-consuming and do not rely on radio chemicals for detection. For both luminescent and fluorescent reporter genes there is a wide variety of options when considering parameters, such as wavelength, co-factor dependency, reporter gene stability, signal intensity and secretable or non-secretable signal. A challenge with the multitude of options is choosing which reporter gene is optimal for a specific application.

Both fluorescent proteins and luciferases have specific advantages and disadvantages. Fluorescent reporters are very bright and can be seen by eye and imaged by means of a fluorescent microscope. Hence, they are often employed in spatio-temporal and visual imaging, as well as in vivo applications^7^. The tandem dimer Tomato (tdTomato) is a genetically improved version of *Discosoma* sp. fluorescent protein (dsRed). tdTomato is an extremely bright red fluorescent protein, being one of the brightest red-shifted fluorescent proteins available and six times brighter than eGFP^8^. One of the main advantages of fluorescent proteins is that they do not require substrates or reagents for detection^9^, however most of them do need oxygen for the formation of their chromophores^10^. Besides, fluorescent proteins can be measured without lysing cells, whereas this is difficult for luciferases. One of the biggest disadvantages of fluorescent reporters is that they need to overcome cellular autofluorescence^11^. However, in general cellular autofluorescence is lower for the emission wavelength of the red channel, favoring a red fluorescent protein such as tdTomato^12^.

Luciferases are proteins that catalyze emission of light from the enzymatic conversion of a supplemented substrate^13^. Hence, the main disadvantage is the need of cofactors when compared to fluorescent reporters. Cofactors such as ATP and metal ions, but also a chemical substrate, are necessary to facilitate the enzymatic reaction that leads to a quantifiable light signal. The most commonly used luciferase is the Firefly luciferase (FFLuc). FFLuc uses ATP, magnesium ions and oxygen as cofactors and the substrate D-luciferin^14^. When measuring in living cells this luminescence reaction requires energy derived from the cell’s metabolism. Therefore, the metabolic state can affect the bioluminescent outcome and induce bias^9^. To circumvent such adverse characteristics, ATP-independent luciferases are now available, such as Nano luciferase (NLuc) and *Gaussia* luciferase (GLuc)^15^.

In contrast to fluorescent reporters, luciferase reporters display low background signal^16^. Aside from auto-fluorescence/luminescence, promoter leakiness also attributes to the background signal of any reporter gene. To decrease potential read-out issues associated with promoter leakiness, unstable reporter genes have been developed^16^. These unstable reporter genes are tagged with a protein degradation signal to facilitate lysosomal breakdown and therefore shorten half-life^17^. This results in very close coupling of transcriptional activity and reporter protein expression and limits potential skewing of results due to promoter leakiness. These unstable reporter variants are employed for both fluorescent and luminescent reporter genes, such as destabilization domain-tdTomato (DD-tdTomato) and Nanoluc-PEST (NLucP)^15,18,19^.

The most commonly used luciferases are expressed intracellularly, and their bioluminescent signal can either be measured in cell lysates or living cells. However, measurements of intracellularly expressed luciferases in living cells can be limited due to factors such as instability of the substrate and low permeability^20–23^. For example, the *Renilla* luciferase substrate has low permeability, whereas the FFLuc substrate is pH-sensitive^21^. Secreted luciferases could be advantageous when performing high-throughput screening or real-time monitoring. GLuc is a naturally secreted luciferase and culture media can therefore be readily used for analysis^24^. While secreted luciferases are suitable for studying the effect of specific molecules on the activity of a selected promoter, this may give issues when studying complex body fluids of unknown composition (e.g. serum or synovial fluid). For example, *Metridia* luciferase (MLuc), another naturally secreted luciferase, was shown to be inactivated in the presence of serum and therefore has limited utility^25^. Nonetheless, the use of GLuc has been reported for in vivo blood and urine studies^26,27^.

In summary, a great variety of different reporter genes is available, however selecting the best reporter gene is challenging and depends on its specific application. Comparative studies have been performed that systematically assess the utility of various reporter genes^28,29^, however these studies are limited in the number of compared reporter genes. In addition, to our knowledge no studies have been reported that compare reporter genes for studying cellular signaling in a complex body fluid environment. Therefore, we systematically investigated the usability of five different reporter genes for pathway activity analyses by using the well-characterized NFκB Response Element (NFκB-RE) and Smad Binding Element (SBE) as model transcription factor binding sites to drive reporter genes^30,31^. We investigated two different stable luciferases, one unstable luciferase, a secretable luciferase and a fluorescent protein and focused on different parameters such as kinetics, inducibility, background signal, sensitivity, potency and compatibility with complex body fluids.

## Methods

### Plasmid construction

Promoter sequences containing either the NFκB-RE or SBE driven by a minimal promoter including a TATA-box (bold) were synthesized by Genecust (Boynes, France)^30,31^. The NFκB-RE DNA fragment contained five NFκB binding sites (underlined): 5′-GGGAATTTCCGGGGACTTTCCGGGAATTTCCGGGGACTTTCCGGGAATTTCCAGATCTGGCCTCGGCGGCCTAGATGAGACACT**AGAGGGTATATAATGGAAGCTCGACTTCCAG**-3′. The SBE DNA fragment consisted of three palindromic SMAD3 binding elements (underlined): 5′-AGTATGTCTAGACTGAAGTATGTCTAGACTGAAGTATGTCTAGACTGACTCGAGGATATCAAGATCTGGCCTCGGCGGCCTAGATGAGACACT**AGAGGGTATATAATGGAAGCTCGACTTCCAG**-3′. These two de novo generated promoter sequences were subsequently cloned into five distinct reporter gene vectors (Table 1). Firstly, KpnI/HindIII (NEB, Beverly, MA) digested NFκB-RE/SBE fragments were ligated with T4 Ligase (NEB) into the KpnI/HindIII digested vectors pNL1.1, pNL1.2, and pGL4.20 (Promega, Madison, WI). Secondly, SacI/HindIII digested NFκB-RE/SBE fragments were ligated into the SacI/HindIII (NEB) digested pDD-tdTomato (Takara Bio, Kyoto, Japan) vector. Lastly, blunted NFκB-RE/SBE fragments were ligated into the *EcoRV* (NEB) digested pGLuc-Basic (NEB) vector and the correct orientation was confirmed. Plasmid DNA was obtained by propagating TOP10 transformed competent cells followed by plasmid DNA extraction with a Plasmid Maxi Kit (Qiagen, Hilden, Germany) according to manufacturer’s protocol. Constructs were validated by Sanger sequencing.

**Table 1:** Reporter gene nomenclature.

Reporter gene	Abbreviation	Vector name
Stable nano luciferase	NLuc	pNL1.1
Unstable nano luciferase	NLucP	pNL1.2
Firefly luciferase	FFLuc	pGL4.20
Gaussia luciferase	GLuc	pGLuc-Basic
tdTomato fluorescent protein	tdTomato	pDD-tdTomato

### Cell culture and transient transfection

SW1353 (ATCC, HTB-94) and HeLa (gift Dr. Jan Willem Voncken, Maastricht University) cells were profiled for short tandem repeat (STR) loci to ensure quality and integrity of the cell lines. Cells were cultured in growth medium consisting of Dulbecco’s Modified Eagle Medium/Nutrient Mixture F-12 with GlutaMAX (DMEM/F12; ThermoFisher, Carlsbad, CA, USA) supplemented with 10% Fetal Calf Serum (FCS; Sigma-Aldrich, Saint Louis, Missouri, USA) and 1% Antibiotic–Antimycotic (ThermoFisher Scientific) in a humidified atmosphere containing 5% CO_2_ at 37 °C. Prior to transfection SW1353 (27,000 cells/cm^2^) and HeLa (18,000 cells/cm^2^) cells were seeded. After overnight attachment of the cells, reporter plasmids were introduced by transient transfection with plasmid DNA using Fugene6 transfection reagent (Promega) according to manufacturer’s instructions. As an internal control for transfection efficiency, pcDNA4/TO/LacZ (ThermoFisher Scientific) was co-transfected, constituting 10% of the total transfected DNA. After 5 h transfection medium was replaced by growth medium. Twenty-four hours post-transfection, the cells were trypsinized (Trypsin; Thermofisher Scientific) and re-seeded into 96-well plates (Greiner Bio-One) at a cell density of 60,000 cells/cm^2^. After 7 h adherence, cells were starved for 16 h prior to stimulation in DMEM/F12 supplemented with 0.1% FCS and 1% Antibiotic–Antimycotic. Serum-starved cells were stimulated with either TGFβ3 (ThermoFisher Scientific) or IL1β (Sigma-Aldrich) with indicated concentrations and period of time in the same medium (*Cf.* Figure legend). Additionally, these recombinant proteins were added in combination with complex body fluids, defined as either 10% osteoarthritis synovial fluid or 10% FCS.

### Synovial fluid and human serum collection

Synovial fluid samples were collected from patients undergoing total knee replacement at the department of orthopedic surgery (Maastricht UMC+). Medical ethical approval for collecting and using synovial fluid was received from the Medical Ethics Committee from the Maastricht University Medical Center (approval number 2017-0183). Dutch medical ethical guidelines were followed for studying human samples and informed consent was acquired. Pure synovial fluid was stored at 4 °C and processed within 6 h. In order to reduce viscosity, pure synovial fluid was diluted three times using DMEM/F12 HEPES (ThermoFisher Scientific). This was followed by centrifugation at 500×*g* for ten minutes at 4 °C. Subsequently, supernatant was transferred to a fresh tube to exclude cell contamination. The supernatant was spun down at 21,380×*g* for 10 min at 4 °C. Finally, supernatant was transferred to fresh tubes and stored at − 80 °C until further use.

Blood was collected from seven healthy volunteers from the internal blood transfusion department (Sanquin, Nijmegen, The Netherlands). All volunteers provided informed consent under institutional ethics committee approved protocols (Commissie Mensgebonden Onderzoeksregio Arnhem—Nijmegen). Blood samples were collected in 8.5 ml BD Vacutainer SST II Advance tubes (BD Vacutainer; Plymouth, UK) and subsequently centrifuged for 10 min at 1000×*g* at 4 °C. Afterwards, the sera were stored at − 80 °C.

### Reporter gene assay

pGL4.20, pNL1.1 and pNL1.2-transfected cells were lysed after stimulation using Cell Culture Lysis Reagent (CCLR; Promega). Nano-Glo (Promega) was used to measure luminescence of the pNL1.1 and pNL1.2 transfected cells. Luminescence of pGL4.20 transfected cells was determined after addition of Luciferase Assay Reagent (Promega). Nano-Glo and Luciferase Assay Reagent were added to 30 μL lysate at 1:1 and 1:2 ratio, respectively. Medium samples were taken from cells expressing the pGLuc-basic plasmid. The amount of luminescence was assessed after the addition of BioLux GLuc Substrate (NEB) in a 1:1 ratio to 30 µL medium sample. All luminescent measurements were performed with the Tristar^2^ LB942 (Berthold Technologies, Bad Wildbad, Germany). pDD-tdTomato expressing-cells were treated with 1 μM shield1 (kindly provided by Dr. Bongers, Radboud University) at the start of stimulation to stabilize the newly produced DD-tdTomato protein. Fluorescent measurements were carried out with a CLARIOstar monochromator (BMG Labtech, Ortenberg, Germany) by exciting the cells at 550 nm and measuring emission at 590 nm. 20 μL of total cell lysate was used for β-Gal colorimetric quantification to correct for differences in transfection efficiency (Invitrogen). Absorbance was determined using a Multiskan FC Microplate Photometer (ThermoFisher Scientific) at 405 nm.

### Gaussia luciferase interference assay

A medium sample was collected 48 h after transfection from SW1353 cells expressing pGLuc-Basic-CMV in serum-free DMEM/F12. This Gaussia luciferase-containing solution was supplemented with either 10% (v/v) FCS, synovial fluid, or human serum. The positive control was similarly diluted with DMEM/F12 instead of a complex body fluid.

### Data analysis

Fold change data from reporter genes measurements were calculated by consecutively subtracting the background signal, correcting for transfection efficiency and normalizing to the mean of the unstimulated control conditions. In addition, concentration–response curves were normalized as percentage from maximal response using the following equation: (X – minimum)/(maximum – minimum) * 100. Potency and maximum fold induction from concentration–response data were calculated using nonlinear regression analysis using GraphPad Prism (version 5.01 for Windows, GraphPad Software, San Diego, CA, USA). The following equation was used to calculate pEC_50_ values: Y = Bottom + (Top–Bottom)/(1 + 10^(LogEC50-X)^).

An unpaired One-tailed Student *t*-test was performed to calculate statistical significance between control and stimulated conditions. Lowest concentration found to be significantly different from control was used to indicate sensitivity. Statistical analysis for inter-donors differences was performed using One-Way ANOVA followed by Bonferroni’s multiple comparison test (GraphPad Prism, version 5.01). All data were presented as mean of tree replicates ± SD, which were considered statistically significant when p ≤ 0.05.

## Results

### Stability of the reporter gene determines temporal resolution

**Figure 1:**
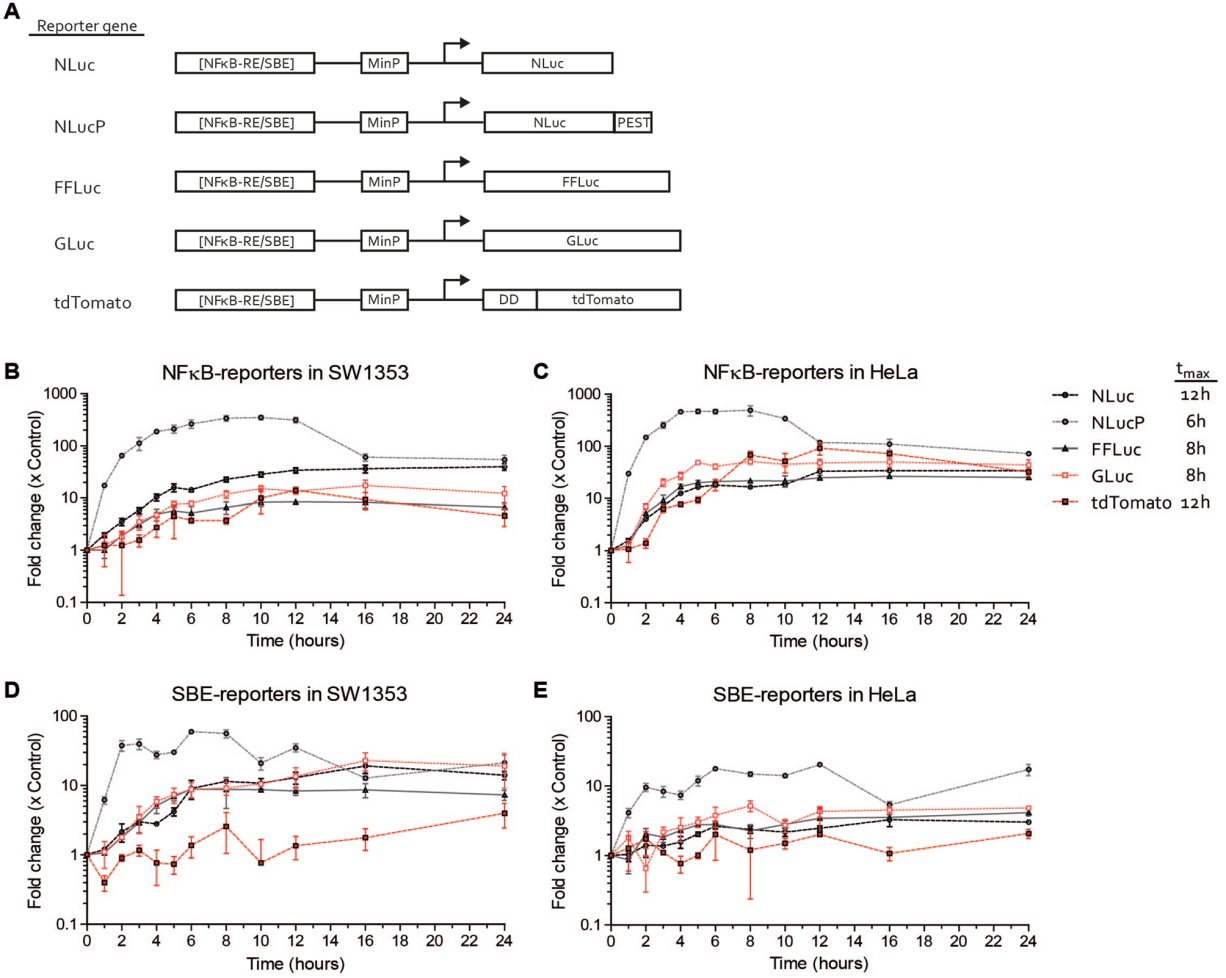
Time-course of five different reporter genes. (**A**) Schematic depiction of expression constructs encoding different types of luciferases and fluorescent reporters driven by Smad binding element (SBE) or NFκB response element (NFκB-RE) promoter. The SBE promoter contains three copies of the SBE and the NFκB promoter contains five copies of the NFκB-RE in front of a minimal promoter (MinP) which includes a TATA-box. The harboring reporter gene is indicated. Time course of (**B**) NFκB-reporters in SW1353 cells (**C**) NFκB-reporters in HeLa cells (**D**) SBE-reporters in SW1353 cells and (**E**) SBE-reporters in HeLa cells. NFκB-reporters were stimulated with 1 ng/mL IL1β and SBE-reporters were stimulated with 1 ng/mL TGFβ3. Average t_max_ of each reporter gene is represented. Data represents mean ± SD of three biological replicates.

In this study, we aimed to establish which reporter gene is optimal for measuring cellular signaling induced by complex body fluids. Therefore, we compared five different reporter genes (NLuc, NLucP, FFLuc, GLuc and tdTomato), individually driven by two different promoter elements (NFĸB-RE and SBE) (Fig. 1A). To determine the kinetics of these different reporter genes, constructed plasmids were transiently transfected in SW1353 and HeLa cells. Reporter gene kinetics in these cell lines were evaluated by stimulating and measuring the responses of the NFĸB-RE and SBE reporters over a period of 24 h with appropriate ligands: IL1β and TGFβ3, respectively (Fig. 1B–E). NFĸB-RE reporters showed a clear induction in both cell lines. The NLucP-NFĸB-RE reporter showed the largest and fastest induction within 6–8 h (Fig. 1B,C). The other three luciferase reporters were clearly induced, but responded slower in reaching maximum induction (8–12 h). In addition, signal of NLuc, FFLuc and GLuc accumulated over time and decreased slightly or remained stable after reaching maximum induction, while the NLucP signal decreased after reaching maximum induction. It took substantially more time before a measurable induction for the fluorescent protein tdTomato was observed as fold induction only emerged above background after 4 h, whereas the other luciferases already revealed this at the first 2 h after stimulation (Fig. 1B,C). Maximum induction for tdTomato was measured at 12 h, after which the tdTomato signal reduced slowly over time. Maximum induction of SBE reporters in both cell lines was lower compared to NFĸB-RE reporters. For example maximum induction in HeLa for NLucP-NFκB-RE was 492 ± 89-fold versus 18 ± 0.3-fold for NLucP-SBE (Fig. 1C,E). However, despite lower inducibility, induction of SBE reporters was clearly observed (Fig. 1D,E). Maximum fold induction of all SBE-dependent reporter genes was higher in SW1353 than in HeLa. For example maximum fold induction of NLucP-SBE was 60 ± 0.9-fold in SW1353 versus 12 ± 1.5-fold in HeLa at 6 h (Fig. 1D,E). In line with the NLucP-NFĸB-RE findings, the NLucP-SBE reporter also exhibited the fastest (within 6 h) and largest response to TGFβ3 stimulation. However, the NLucP-SBE reporter had a different kinetic pattern with a drop in induction after 2–3 h (Fig. 1D,E). The other three luciferase reporters demonstrated a slower accumulating induction without a detectable signal drop. Induction of tdTomato-SBE signal was not detected in both cell lines, with values fluctuating close to background levels (Fig. 1D,E). Next, the time of maximum induction (t_max_) was determined for every reporter gene, which was defined as the latest time point before reaching a plateau value based on the kinetic responses of SBE and NFĸB-RE in SW1353 and HeLa cells. This resulted in the following t_max_ characteristics: stable Nano luciferase (NLuc; t_max_ = 12 h), unstable Nano luciferase (NLucP; t_max_ = 6 h), Firefly luciferase (FFLuc; t_max_ = 8 h), *Gaussia* luciferase (GLuc; t_max_ = 8 h) and tdTomato (t_max_ = 12 h) (Fig. 1). To determine if the here observed induction of luciferase or fluorescent activity reflects the actual mRNA transcriptional activity of the reporter constructs, the NFκB-RE reporters were stimulated with IL1β for 2 h. Luciferase activity or fluorescence were then determined, as well as mRNA levels of the individual reporter genes. A similar pattern was observed for the reporter luciferase/fluorescent activity versus their corresponding mRNA levels (Supplemental Figure 1A,B). NLucP presented with both the highest fold induction at the luciferase activity level as well as at mRNA level. In conclusion, NLucP, as a reporter gene, showed in all conditions the steepest and highest fold inducibility, while the fluorescent protein tdTomato responded slowest and delivered lowest inducibility compared to all other tested luciferase reporter genes.

### Sensitivity and potency of reporter genes differs among cell lines and response elements

**Figure 2:**
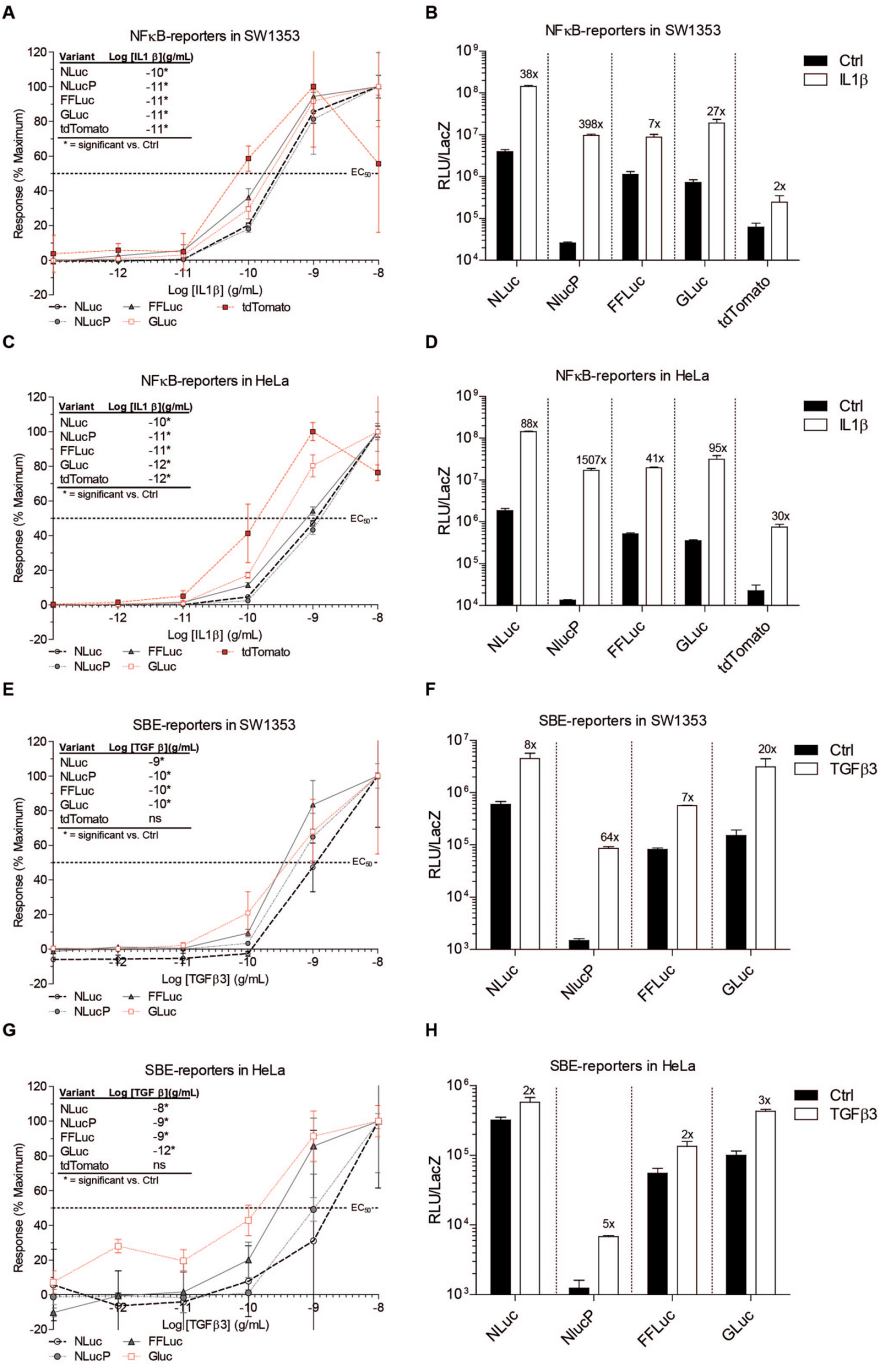
Concentration–response curve of five different reporter genes. Response was determined at t_max_ of each specific reporter system. NFκB-RE and SBE reporters were stimulated with IL1β (10^–8^–10^–13^ g/mL) and TGFβ3 (10^–8^–10^–13^ g/mL), respectively. Maximum response was set at 100% and total counts are shown (for all luciferase reporter genes at a concentration of 10^–8^ g/mL of either IL1β or TGFβ3 and for tdTomato at a concentration of 10^–9^ g/mL IL1β). Sensitivity is represented as lowest concentration ligand that could be detected significantly compared to control condition. Concentration curve, sensitivity and total counts of (**A**,**B**) NFκB-reporters in SW13 cells (**C**,**D**) NFκB-reporters in HeLa cells (**E**,**F**) SBE-reporters in SW1353 and (G-H) SBE-reporters in HeLa. Data represents mean ± SD of three biological replicates. ns, not significant. N.D. Not Detected.

Next, we investigated the sensitivity of the different reporter genes by determining ligand concentration–response curves. Sensitivity was defined as the lowest concentration of ligand that could be detected with statistical significance using the reporters. All NFĸB-RE-driven reporters responded in an IL1β concentration-dependent manner (Fig. 2A–D). In SW1353 cells, NLucP, FFLuc, GLuc and tdTomato were most sensitive (10^–11^ g/mL) and NLuc was least sensitive (10^–10^ g/mL). In HeLa cells, GLuc and tdTomato were the most sensitive (10^–12^ g/mL), followed by NLucP and FFLuc (10^–11^ g/mL) and NLuc was the least sensitive (10^–10^ g/mL). Potency (pEC_50_), defined as the concentration at which 50% of the response is measured, was relatively similar between luciferases. However, tdTomato-NFĸB-RE was found to be the most potent (Table 2). Count number revealed that background counts for the NLucP were extremely low compared to the other luciferase variants at the highest tested IL1β concentration (Fig. 2B,D). Therefore, and in accordance with the kinetic measurements, NLucP-NFĸB-RE exhibited at least a tenfold higher induction capacity compared to the other reporters, with a maximum of 397.7 ± 20.5 in SW1353 and 1507 ± 57.9 in HeLa (Fig. 2B,D; Table 2). FFLuc demonstrated the lowest induction of the luciferases, with a maximum of 7.0 ± 0.33 in SW1353 and 41.1 ± 0.65 in HeLa (Table 2). Even though background counts were low, tdTomato-NFĸB-RE revealed moderate induction in the HeLa cell line (maximum 29.7-fold), whereas in SW1353 cells it was the least induced reporter system (maximum 2.1-fold) (Fig. 2B,D). For the SBE reporters, all luciferase reporters displayed a TGFβ3 concentration-dependent response curve, however tdTomato lacked responsiveness and a concentration–response curve could not be obtained (Fig. 2E,G). In SW1353 cells, the SBE-driven luciferases, NLucP, FFLuc and GLuc were equally sensitive (10^–10^ g/mL). Again NLuc revealed to be the least sensitive reporter gene (10^–9^ g/mL). No significant inductions were determined for tdTomato with any of the tested TGFβ3 concentrations in either cell lines. In HeLa cells, GLuc-SBE was the most sensitive (10^–12^ g/mL), followed by NLucP and FFLuc (10^–9^ g/mL) and NLuc (10^–8^ g/mL). In addition, potency did not differ between SBE reporter genes in SW1353 cells, however in HeLa cells there were some little differences in potency (Table 2). In HeLa cells, GLuc was most potent, followed by FFLuc, NLucP and NLuc. When looking closer to the number of counts, no induction was observed with the tdTomato-SBE reporter in either cell line (Fig. 2F,H). Overall, SBE reporters in HeLa cells revealed a small induction compared to SBE-reporters in SW1353 (Fig. 2F,H). In SW1353 and HeLa cells, NLucP provided the highest fold induction (Fig. 2F,H). In summary, sensitivity differed among cell lines and response elements. Furthermore, potency between reporter genes did not differ greatly for both NFκB-RE and SBE transcription factor binding elements. However, background counts for NLucP were in all conditions extremely low and, resulting in the largest fold induction observed for all reporter genes that we tested. Based on fold induction, tdTomato was inferior to the other reporter genes, despite being the most potent NFκB-RE reporter gene.

**Table 2:** Potency and maximum fold induction of NFκB-RE and SBE reporters.

Response element	Reporter gene	pEC_50_		Fold induction	
		SW1353	HeLa	SW1353	HeLa
*NFκB-RE*	*NLuc*	9.6 ± 0.05	9.0 ± 0.04	37.8 ± 1.09	87.5 ± 1.21
	*NLucP*	9.5 ± 0.08	8.9 ± 0.06	397.7 ± 20.5	1507 ± 57.9
	*FFLuc*	9.8 ± 0.07	9.1 ± 0.03	7.0 ± 0.33	41.1 ± 0.65
	*GLuc*	9.7 ± 0.09	9.5 ± 0.08	27.0 ± 1.49	95.2 ± 5.31
	*tdTomato*	10.1 ± 0.22	9.9 ± 0.11	2.1 ± 0.63	29.7 ± 2.54
*SBE*	*NLuc*	9.0 ± 0.12	8.8 ± 0.28	8.1 ± 0.73	1.1 ± 0.42
	*NLucP*	9.2 ± 0.07	9.0 ± 0.06	63.9 ± 3.05	5.2 ± 0.14
	*FFLuc*	9.4 ± 0.08	9.60 ± 0.14	6.5 ± 0.32	1.6 ± 0.13
	*GLuc*	9.4 ± 0.16	10.0 ± 0.13	20.2 ± 2.57	2.8 ± 0.20
	*tdTomato*	N.D	N.D	0.9 ± 1.06	1.0 ± 0.96

Potency (pEC_50_) and maximum fold induction were determined from a concentration–response experiment at t_max_ of each specific reporter system. NFκB-RE and SBE reporters were stimulated with IL1β (10^–8^–10^–13^ g/mL) and TGFβ3 (10^–8^–10^–13^ g/mL), respectively. The presented values were calculated with Graphpad Prism analysis using nonlinear regression: Log(Agonist) vs Response. Data represents mean ± SD of three biological replicates. N.D. Not determined.

### Secreted luciferase signal is strongly reduced by complex body fluids

**Figure 3:**
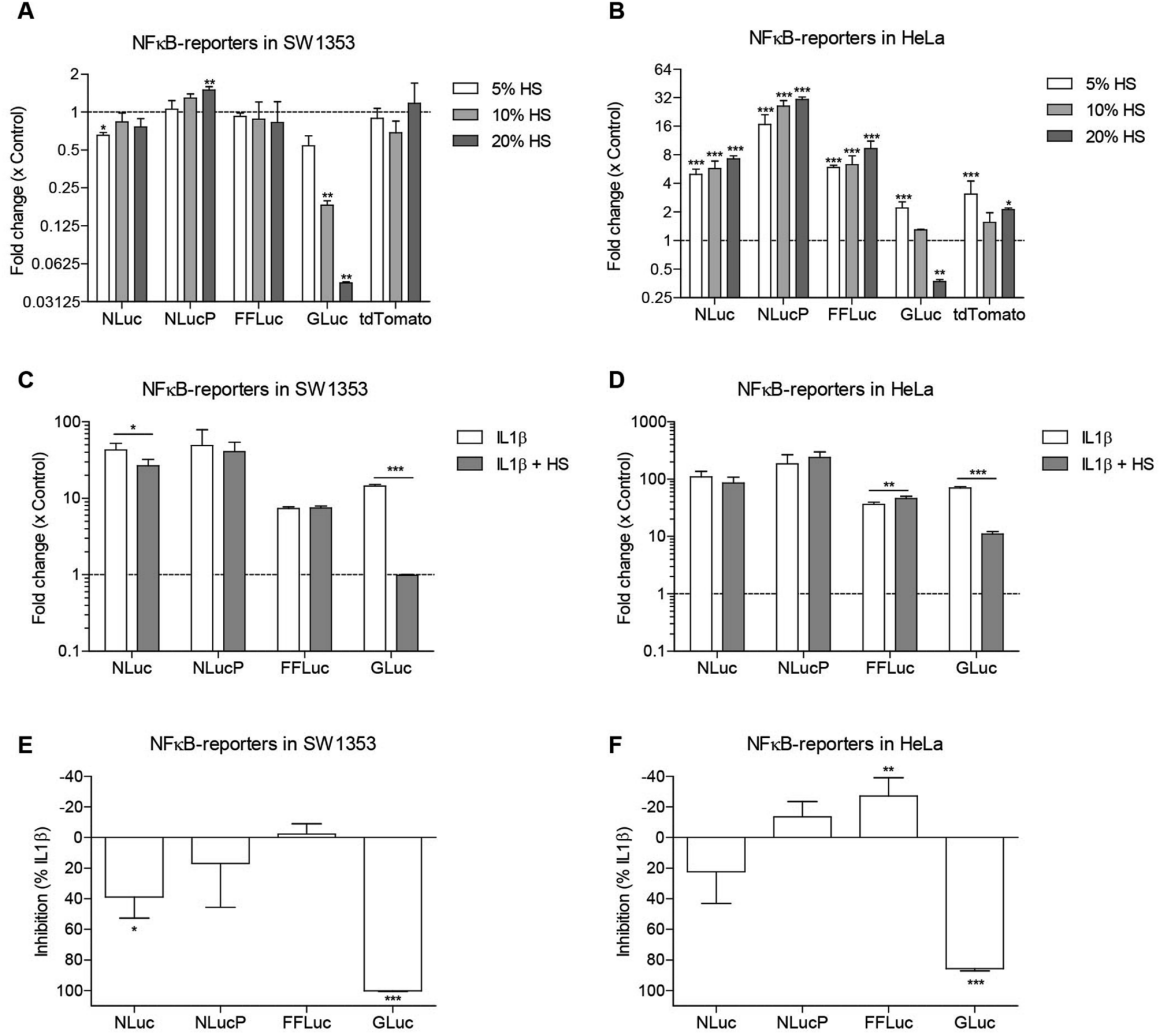
Human serum interference with secreted Gaussia luciferase. Fold change was determined at t_max_ of each specific reporter system. NFκB-RE reporters were stimulated with different concentrations of HS (5%,10% or 20%) in (**A**) SW1353 cells and (**B**) HeLa cells. NFκB-RE reporters were stimulated with IL1β (1 ng/mL) with or without supplementation of 10% HS. Fold change of (**C**) NFκB-reporters in SW1353 cells and (**D**) NFκB-reporters in HeLa cells. Percentage inhibition of stimulation with IL1β in combination with HS compared to IL1 β stimulation alone was determined in (**E**) SW1353 cells and (**F**) HeLa cells. HS; Human Serum. Data represents mean ± SD of four biological replicates. **p. value ≤ 0.01,***p. value ≤ 0.001.

We investigated the sensitivity of the different reporter genes in a model system; i.e. to known positive stimuli (IL1β and TGFβ3). To investigate if the sensitivity of the different reporter genes is affected by complex body fluids, we tested responsiveness of the NFĸB-RE reporters to various concentrations of human serum (HS), fetal calf serum (FCS) or synovial fluid (SF) (Fig. 3; Supplemental Figure 2). As expected, for all reporter genes in both cell lines the fold induction was much lower with a complex body fluid compared to known positive stimuli (Fig. 3A,B; Supplemental Figure 2A,D). Furthermore, fold inductions were more evident in HeLa cells compared to SW1353 cells (e.g. maximum 1.5-fold in SW1353 and 30.8-fold in HeLa for HS). In SW1353 cells, only NLucP demonstrated a significant fold induction upon stimulation with the highest concentration HS (20%) (Fig. 3A), indicating that NLucP is the most sensitive reporter in this context. No significant changes for NLuc, FFLuc and tdTomato were detected with any of the tested concentrations HS. Surprisingly, GLuc demonstrated a significant decrease for both 10% and 20% HS. In HeLa cells, NLuc, NLucP and FFLuc displayed significant fold inductions for all tested concentrations HS in a concentration-dependent manner (Fig. 3B). Here, NLucP also exhibited highest sensitivity as indicated by the highest fold induction with all tested concentrations HS. Interestingly, GLuc displayed a significant fold induction with 5% HS, whereas with higher concentration of HS the signal was equal or lower than control. The tdTomato also demonstrated a significant fold induction for both 5% and 20% HS, whereas no significant fold induction could be detected for 10% HS. For the other complex body fluids tested, FCS and SF, GLuc demonstrated this same pattern, where a higher concentration of body fluid resulted in lower fold inductions (even below control condition) compared to the lower concentrations of body fluid tested (Supplemental Figure 2A,D). This is in clear contrast with the NLuc, NLucP, FFLuc and tdTomato reporter genes that displayed either no induction or responded in a concentration-dependent fashion.

To investigate if this reduction effect of GLuc is the result of the compatibility of this reporter gene with complex body fluids, we tested the responsiveness of the NFĸB-RE reporters to IL1β alone, or in combination with HS, FCS or SF (Fig. 3C,F; Supplemental Figure 2E,L). The tdTomato reporter was not investigated in these conditions, since it became evident that the tdTomato reporter gene exhibited an unfavorable fold induction. A very strong significant reduction of the IL1β-induced luciferase signal from GLuc-NFκB-RE was observed in both SW1353 and HeLa cells stimulated in combination with a complex body fluid (Fig. 3C,D; Supplemental Figure 2E,H). To determine the impact of complex body fluids on the luciferase signal, we calculated the percentage inhibition of IL1β in combination with complex body fluid compared to IL1β stimulation alone. HS impacted the signal in the most prominent manner: the fold change of the GLuc signal was reduced to approximately background values (100% reduction compared to IL1β induction alone in SW1353 and 86% in HeLa; Fig. 3E,F). In addition, FCS and SF also inhibited the IL1β-induced GLuc signal when compared to IL1β stimulation alone, with FCS 55% reduction in SW1353 and 27% in HeLa and SF causing a 89% reduction in SW1353 and 76% in HeLa (Supplemental Figure 2I,L). For the intracellularly measured luciferases the signal reduction caused by HS, FCS or SF was much lower and NLucP and FFLuc even showed a small increase in signal upon stimulation with some of the complex body fluids. The luciferase signal from NLuc was most reduced of the tested intracellular luciferases (e.g. 29% in SW1353 and 22% in HeLa by FCS; Supplemental Figure 2I,J), however only the signal reduction caused by HS stimulation was significant in SW1353 (39% in SW1353 by HS; Fig. 3E), while all the other reductions were not significant (Fig. 3E,F; Supplemental Figure 2I,L). NLucP was also reduced (e.g. 10% in SW1353 and 12% in HeLa by SF; Supplemental Figure 2K,L), however all not significant and in HeLa a small non-significant increase in signal was observed when stimulated with either HS or FCS (− 14% by HS and − 9% by FCS; Fig. 3F and Supplemental Figure 2J). Stimulation with HS in HeLa cells and stimulation with SF in SW1353 cells resulted in a significant increase of FFLuc signal (Fig. 3F; Supplemental Figure 2K), whereas non-significant increases of FFLuc signal were observed with the other body fluids in both cell lines (e.g. − 6% in SW1353 and − 10% in HeLa by FCS; Supplemental Figure 2I,L).

### Reduction of secreted luciferase signal by complex body fluids is donor-dependent

**Figure 4:**
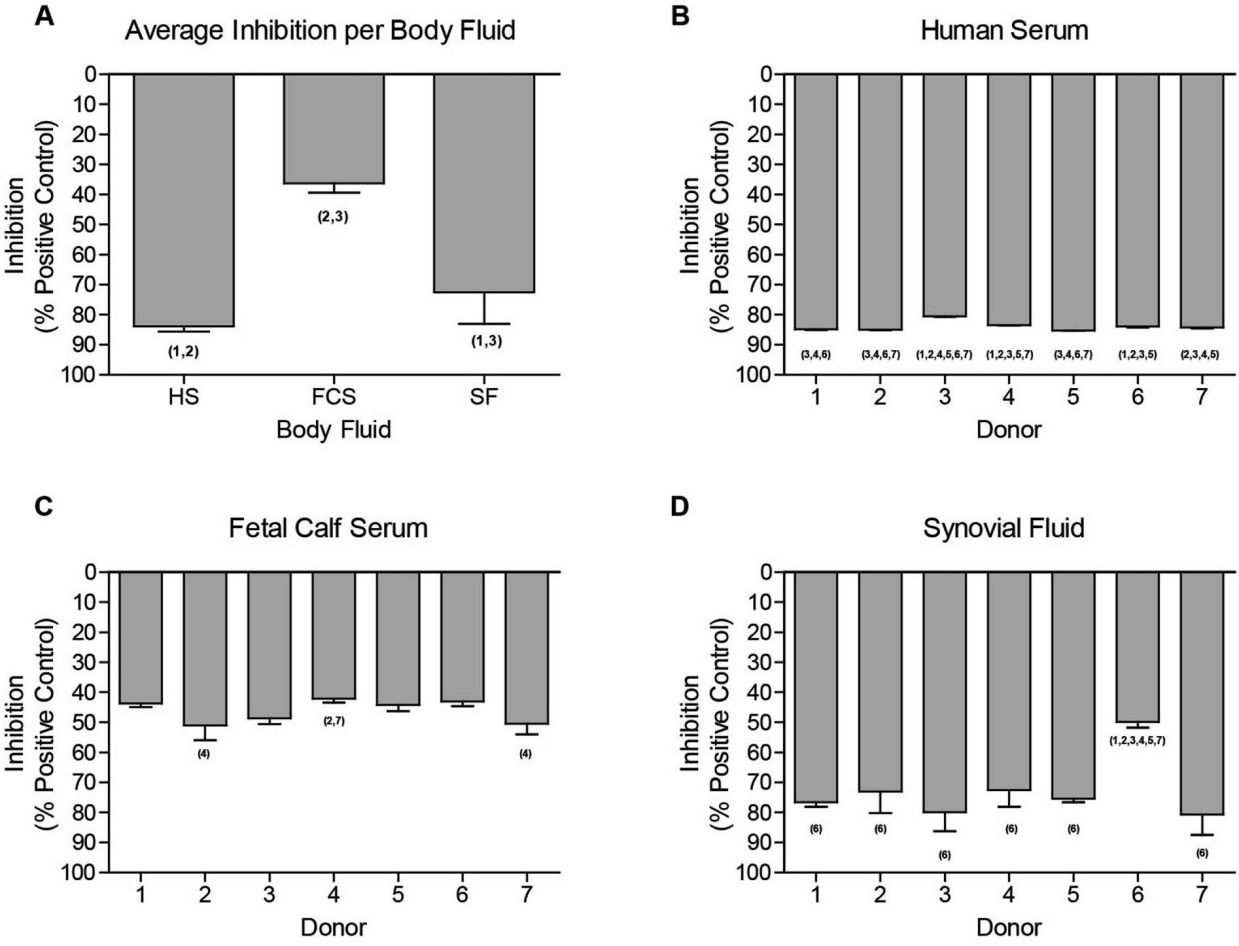
Inhibition of GLuc activity by individual body fluid donors. Different kinds of body fluids were added to the GLuc containing medium sample to a final concentration of 10%. (**A**) The average inhibition of each body fluid. Seven donors of (**B**) HS, (**C**) FCS and (**D**) HS were studied for their individual effect on inhibition of the GLuc signal. Control condition (medium sample of SW1353 cells transfected with CMV-GLuc) was equally diluted with serum-free DMEM/F12. FCS; Fetal Calf Serum, SF; Synovial Fluid, HS; Human Serum. Data shown represents mean ± SD of three technical replicates. Statistical differences are indicated with the donor number that significantly is different (p ≤ 0.05).

Since GLuc was heavily inhibited by complex body fluids, we next evaluated whether this was (1) directly caused by interference of the GLuc enzyme by the complex body fluid, (2) whether inhibition depends on the type of body fluid, and (3) whether this differs between individual donors of such body fluids. To address these questions we exposed a secreted GLuc-containing medium sample to HS, FCS or SF from seven different donors (Fig. 4). The luciferase signal in the Gluc-containing medium was set at maximum signal intensity. Substantial inhibition of the GLuc signal was observed with all body fluids tested (HS, FCS and SF) for all individual donors. HS inhibited the GLuc signal the most pronounced (84% ± 1.5), SF second (72% ± 10.5) and FCS inhibited the signal the least (46% ± 4.1; Fig. 4A). Importantly, we found significant differences in the magnitude of inhibition between donors, with the largest variability observed amongst SF donors (Fig. 4B–D; Supplemental Table 1). Additionally, activity of other kind of secreted luciferases, such as secreted Nano and *Cypridina* luciferases, was also affected by the presence of specific body fluids (Supplemental Figure 3).

**Figure 5:**
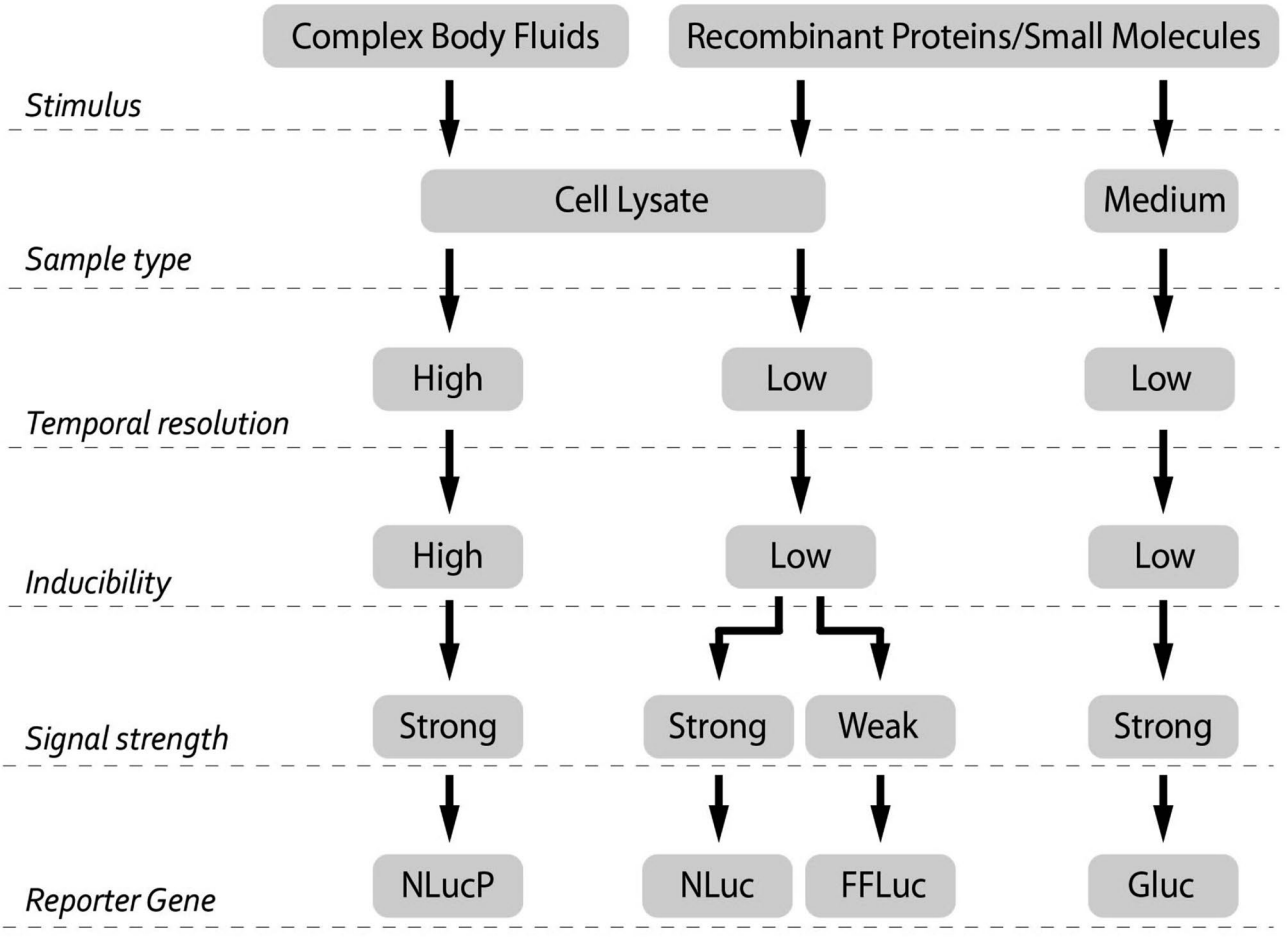
Flow chart: recommended applications of the different reporter genes for specific experimental designs. A recombinant protein or small molecule can be studied in both medium as well as cell lysate. Complex body fluids is preferably studied with cell lysates. Unstable nature of NLucP enables higher temporal resolution for monitoring transcriptional activity compared to stable accumulating luciferases (NLuc, FFLuc, GLuc). Furthermore, NLucP exhibits substantial higher inducibility than other luciferases. Among luciferases, FFLuc displays the lowest signal strength.

Together, this study shows that the choice of reporter gene is very important, especially when measuring responses in and by complex body fluids. This choice should take into account parameters such as stimulus, sample type, temporal resolution, inducibility and signal strength. Based on our findings we designed a decision tree that can be used to determine which of the reporter genes used in this study is best to use in a certain experimental setting (Fig. 5).

## Discussion

The use of reporter gene assays has been evolving. Multiple applications have been identified, such as transcriptional activity studies, imaging studies, and molecular pathway studies^29,32,33^. For these purposes many variants of fluorescent and luminescent reporter genes have been used. In addition, reporter genes have also been described as a valuable tool to study complex body fluids, *e.g.* urine and blood^34–36^. However, there is a very limited body of literature on the compatibility of different reporter genes to study complex body fluids. In addition to compatibility, a considered selection of a reporter gene based on unique characteristics can be a major determinant for study outcome. Here, we compared five different reporter genes to determine their performance for monitoring cellular signaling in a complex body fluid environment. Performance was assessed by a number of criteria including kinetics, potency (i.e. pEC_50_), sensitivity (i.e. lowest concentration that gives significant response), detection window (i.e. maximum inducibility), and body fluid compatibility. We found that the tdTomato fluorescent reporter gene demonstrated limited or lack of inducibility. Importantly, the secreted GLuc signal was strongly impacted by the presence of complex body fluids, yielding uninterpretable results. Both stable and unstable NLuc demonstrated clear inducibility and were compatible with body fluid stimulation. Only NLucP visibly revealed temporal signaling. The widely used FFLuc was compatible with complex body fluids. However, it had similar or inferior responses in comparison to stable NLuc.

Five reporter genes driven by two well-described response elements, NFκB-RE and SBE, were studied in two commonly used cell lines. The response of these reporter genes to IL1β or TGFβ3 was monitored over a period of 24 h, yielding distinct response dynamics. The most obvious finding was that the NLucP reporters demonstrated rapid and highest inductions for both response elements in both cell lines. This was accompanied with the lowest signal stability over time, as the signal decreased quickly after reaching maximum induction. Due to its unstable nature, the NLucP is suitable for monitoring rapid signal fluctuations in time. For example, the fluctuation in TGFβ-induced SBE activity from 2 to 6 h of stimulation follows the reported fluctuation in receptor-activated SMAD phosphorylation^37^. Destabilization of Firefly luciferase by the addition of a PEST domain similar to NLucP has been described by Robertoson et al*.* to increase inducibility by lowering basal luciferase activity and allow detection of transcriptional activity in time^38^. NLuc revealed a robustly accumulating signal over time, where other reporter genes, like FFLuc, tdTomato and GLuc, demonstrated equal or inferior signal stability. In addition, the signal intensity of FFLuc was found to be lower when compared to Nano luciferases^15,39^. Interestingly, tdTomato driven by the SBE lacked induction in both SW1353 and HeLa cells. HeLa cells are known to be minimally responsive to TGFβ-induced SBE activation^40^. In SW1353, this is might be explained by the weaker activation by SBE in combination with cellular autofluorescence, whereby autofluorescence is known to mask any positive signal as was described for GFP^11^. The tdTomato as a reporter gene demonstrated slower induction characteristics compared to luciferase reporters. This finding is consistent with a number of studies that demonstrated slower kinetics and sensitivity for fluorescent proteins (i.e. Ypet, GFP, DsRed) when compared to Firefly luciferases^9,29,41^. Unlike luciferases, fluorescent proteins require maturation after folding, resulting in slower kinetics^42,43^. Furthermore, it has been demonstrated that fluorescent proteins with shorter maturation times reveal greater signal-to-noise ratios and temporal resolution^43^.

We confirmed differences in the magnitude of induction between reporter genes with ligand concentration–response curves. NLucP as a reporter gene driven by NFκB-RE demonstrated at least a tenfold higher induction compared to the other NFκB-RE-driven reporter genes tested. However, if not compared to the NLucP, the other three luciferase reporter genes still revealed clear inducibility. In contrast, NFκB-RE-driven tdTomato demonstrated lower inducibility, however appeared to be most potent (i.e. highest pEC_50_) when tested in HeLa cells. A possible explanation for this might be that tdTomato stabilization occurs by Shield1, which was added simultaneously with stimulation, leading to stronger signal accumulation. This was not the case for tdTomato-driven by SBE, where any detectable induction of tdTomato levels remained absent. Potency and sensitivity between luciferases did not differ greatly, although it should be noted that GLuc tended to be the most potent and sensitive reporter gene. This might be explained by the fact that upon stimulation the medium was replaced and any produced and secreted GLuc was removed, thereby potentially improving signal over background. Notably, the choice for a reporter gene may be particularly critical if it is driven by a weak promoter element. For instance, tdTomato was only clearly detectable when driven by the strong response element NFκB and measured in IL1β-sensitive HeLa cells, whereas a weaker response element, like SBE, resulted in a complete lack of detectability. This particular difference in transcriptional activation between NFκB-RE and SBE might be due to the different amount of transcription factor binding sites, as NFκB-RE consists of five repeats, while SBE only contains three. Similar to tdTomato, it has been described that GFP requires strong promoter activity for detection and is not suitable for studying weak promoters^44^. In contrast, NLucP driven by SBE was capable of detecting TGFβ3 responses in HeLa cells, which are minimally responsive to TGFβ-induced SBE activation, indicating its potential use for studying slight changes in cellular signaling^40^.

Next we investigated whether these different reporter genes are compatible with complex body fluids. For this purpose, the NFκB-RE-driven luciferase reporters were used. First, a concentration series of body fluids were investigated to determine sensitivity and compatibility. Interestingly, only the NLucP demonstrated concentration-dependent induction upon stimulation with body fluids (HS, FCS and SF) in SW1353 cells, which again emphasized the sensitivity of this particular reporter gene. Moreover, in HeLa cells all luciferases except GLuc displayed a clear concentration-dependent induction. Strikingly, GLuc exhibited a concentration-dependent decreases in signal. Secondly, body fluids spiked with IL1β were tested for their performance compared to IL1β alone. The IL1β-induced signal of the intracellularly measured luciferases (i.e. NLuc, NLucP, FFLuc) was influenced to a certain extent when cultures were supplemented with HS, FCS or SF. We expect that this may have resulted from signaling cross-talk induced by the body fluids, which contain a large set of signaling molecules that can activate other pathways that might interact with NFκB signaling. Repeatedly, the GLuc signal was substantially inhibited when synovial fluid or FCS were combined with IL1β stimulation. In particular, SF and HS strongly reduced the GLuc signal to almost background levels. GLuc is secreted into the medium by the reporter cell and this medium is subsequently used for the quantification of luminescence. Interference of luciferase activity by blood or serum have been reported in literature^25,45^. However, to our knowledge, interference with GLuc has never been reported, while it is extensively used for in vivo studies in which a serum component is evident^46,47^. The presence of a complex body fluid might interfere with the bioluminescent reaction by affecting either the enzymatic activity of the secreted luciferase or the bioluminescent substrate. Therefore we performed a direct comparison of the same GLuc sample spiked with either HS, FCS, or SF each derived from seven individual donors. Without exception, the combination of these body fluids with GLuc led to substantial reduction of the basal GLuc activity. The strongest interference was observed for SF and HS. Although the signal interference is a major concern in itself, more important are the differences in interference magnitude between donors of the same type of body fluid. Inter-donor variation for interference with GLuc was clearly observed (*Cf.* Fig. 4). As a consequence, cellular signaling responses provoked by body fluids (of the same type) from different donors cannot be compared to each other using Gluc as a reporter gene, as they might differ in magnitude of GLuc inhibition. Similar to GLuc, secreted Nano- and Cypridina luciferase demonstrated an altered activity in the presence of specific body fluids. An earlier study reported altered bioluminescent reactions for the secreted Nano luciferase with different cell culture media, such as DMEM, RPMI1640, D10 and R10^48^. Moreover, it has been demonstrated that the pH and the presence of FCS might affect the bioluminescent reaction of secreted NLuc^48^. Our data confirm an increase in bioluminescent signal upon addition of FCS. Based on these findings, it is advised to avoid using secreted GLuc for studying cellular responses in the presence of complex body fluids, as this could lead to misinterpretation of results. Hence, we want to emphasize that the choice of reporter gene is essential, especially when measuring complex body fluid responses. This choice should take into account parameters such as stimulus, sample type, type of measurement, inducibility and signal strength. An overview of characteristics and recommended use of the different reporter genes is presented in Fig. 5.

In summary, when conducting a promoter assay, the choice of the reporter gene has a great influence on the experimental outcome. Sensitivity, stability and inducibility differ greatly between reporter genes and this aspect should be taken into account when choosing a reporter gene. The fluorescent reporter tdTomato can be a valuable tool for monitoring intracellular signaling or in vivo applications. However, it demonstrated important limitations, as it required strong transcriptional activation for detection and exhibited slow kinetics. NLucP showed high sensitivity paired with a large detection window and is therefore suitable for studying weaker promoters or detection of subtle changes in cellular signaling. Additionally, it is a useful tool to monitor transcription high temporal resolution. The stable reporters, Nano-, Firefly-, and *Gaussia* luciferase exhibited lower inducibility, however they provided well detectable and quantifiable responses. The three luciferases measured in cell lysates displayed compatibility with complex body fluids, whereas the signal of secreted GLuc was quenched in the presence of complex body fluids. Of even greater concern were the substantial inter-donor differences in the degree of GLuc activity inhibition. Therefore, when considering secreted luciferases for studying complex body fluids, compatibility should be carefully assessed beforehand. Hence, we found that NLucP is currently the most useful for studying complex body fluids.

## Supplementary Information

Supplementary Information 1.

Supplementary Information 2.

Supplementary Information 3.

Supplementary Information 4.

Supplementary Information 6.
