# Supplementary material for: Reporter gene comparison demonstrates interference of complex body fluids with secreted luciferase activity
Source: Sci Rep. 2021 Jan 14;11:1359. doi: 10.1038/s41598-020-80451-6 (PMC7809208; doi:10.1038/s41598-020-80451-6)
Supplement: Supplementary file 1 — Supplementary Information 1. [file 41598_2020_80451_MOESM1_ESM.docx]

**
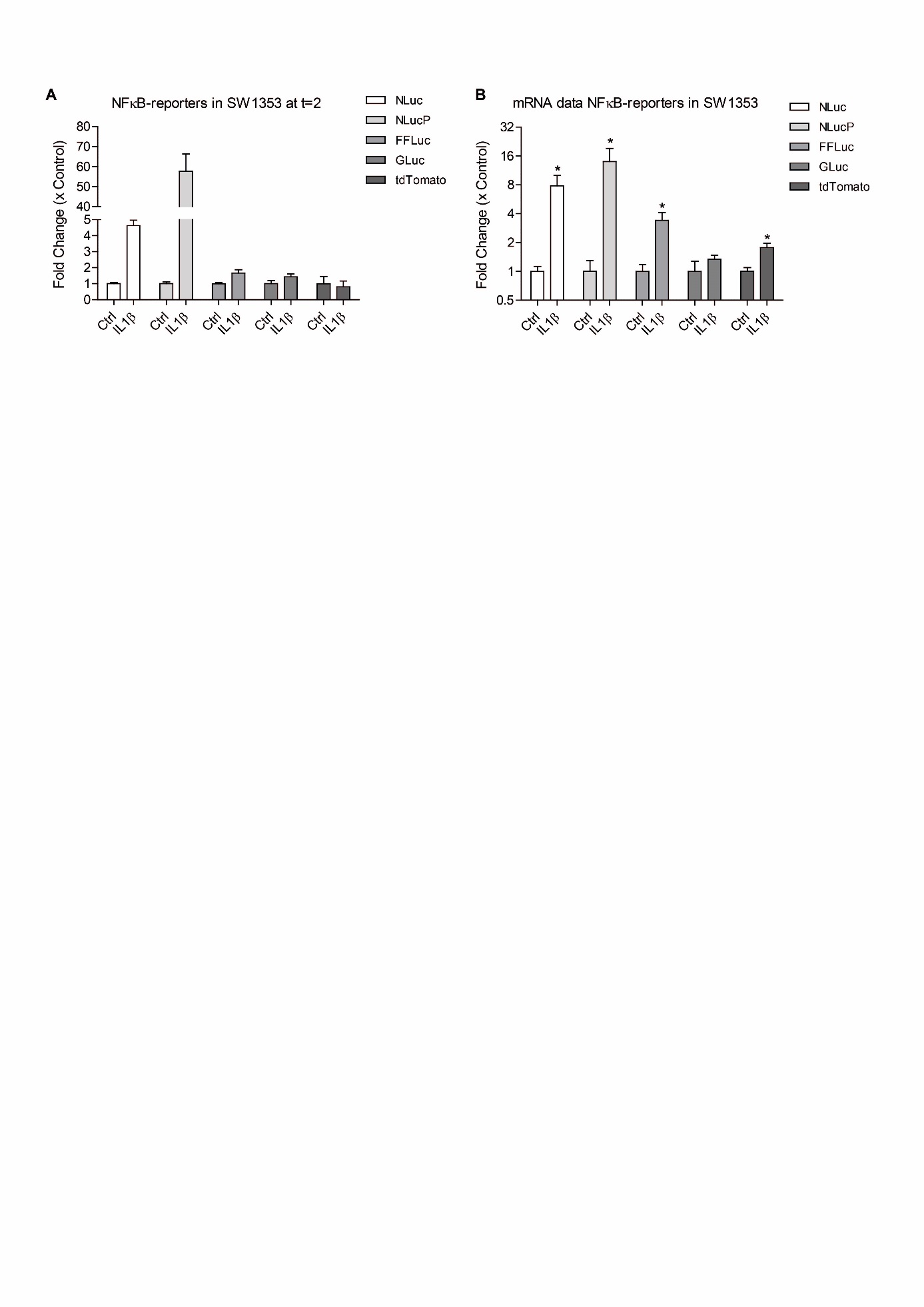
**

**Supplementary Figure 1**. NFκB-RE reporters were stimulated with 1 ng/mL IL1β for 2 hours in SW1353 cells for (A) reporter gene level measurement and (B) mRNA levels. Data represents mean ± SD of three biological replicates.
