# Supplementary material for: Reporter gene comparison demonstrates interference of complex body fluids with secreted luciferase activity
Source: Sci Rep. 2021 Jan 14;11:1359. doi: 10.1038/s41598-020-80451-6 (PMC7809208; doi:10.1038/s41598-020-80451-6)
Supplement: Supplementary file 2 — Supplementary Information 2. [file 41598_2020_80451_MOESM2_ESM.docx]

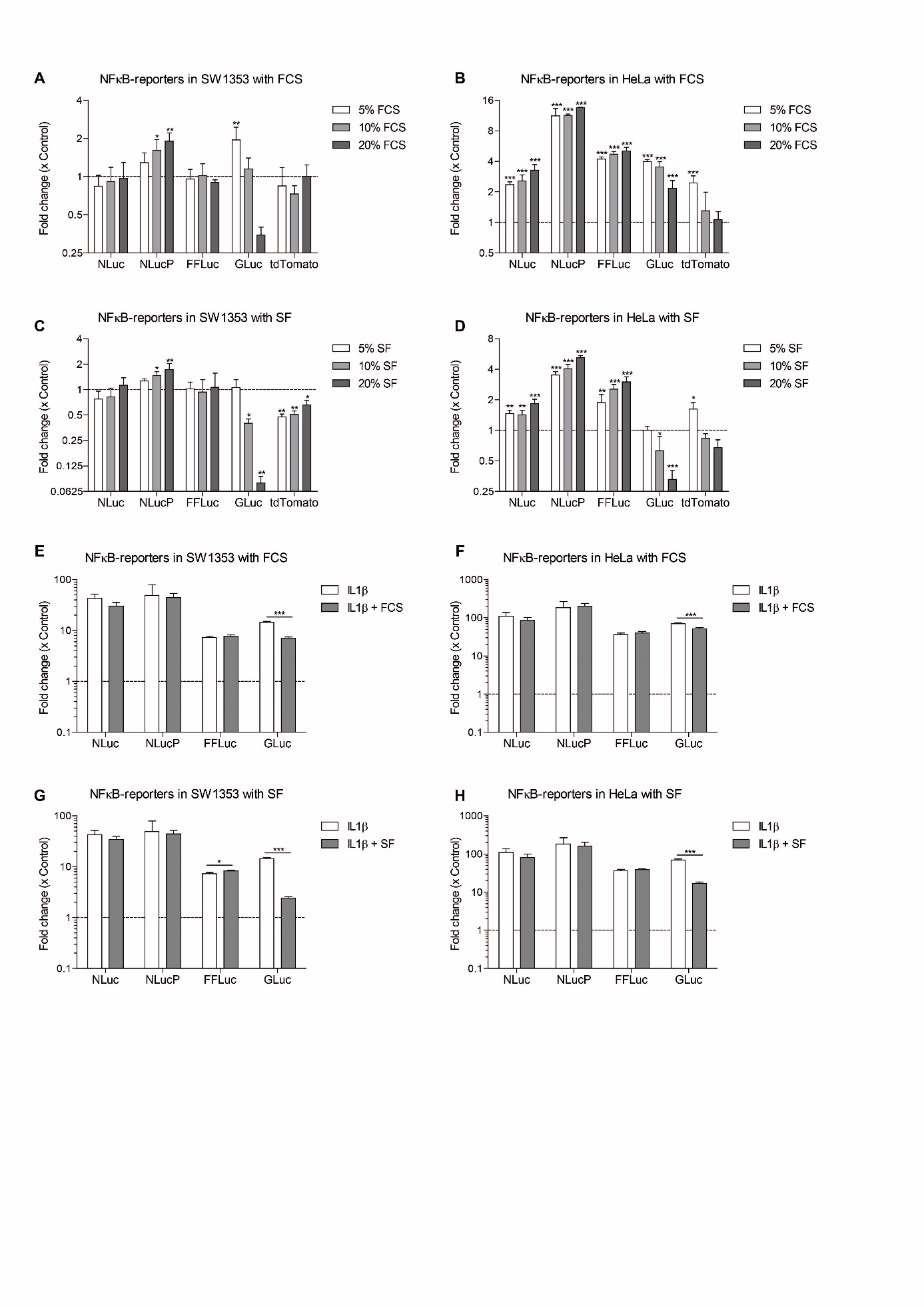


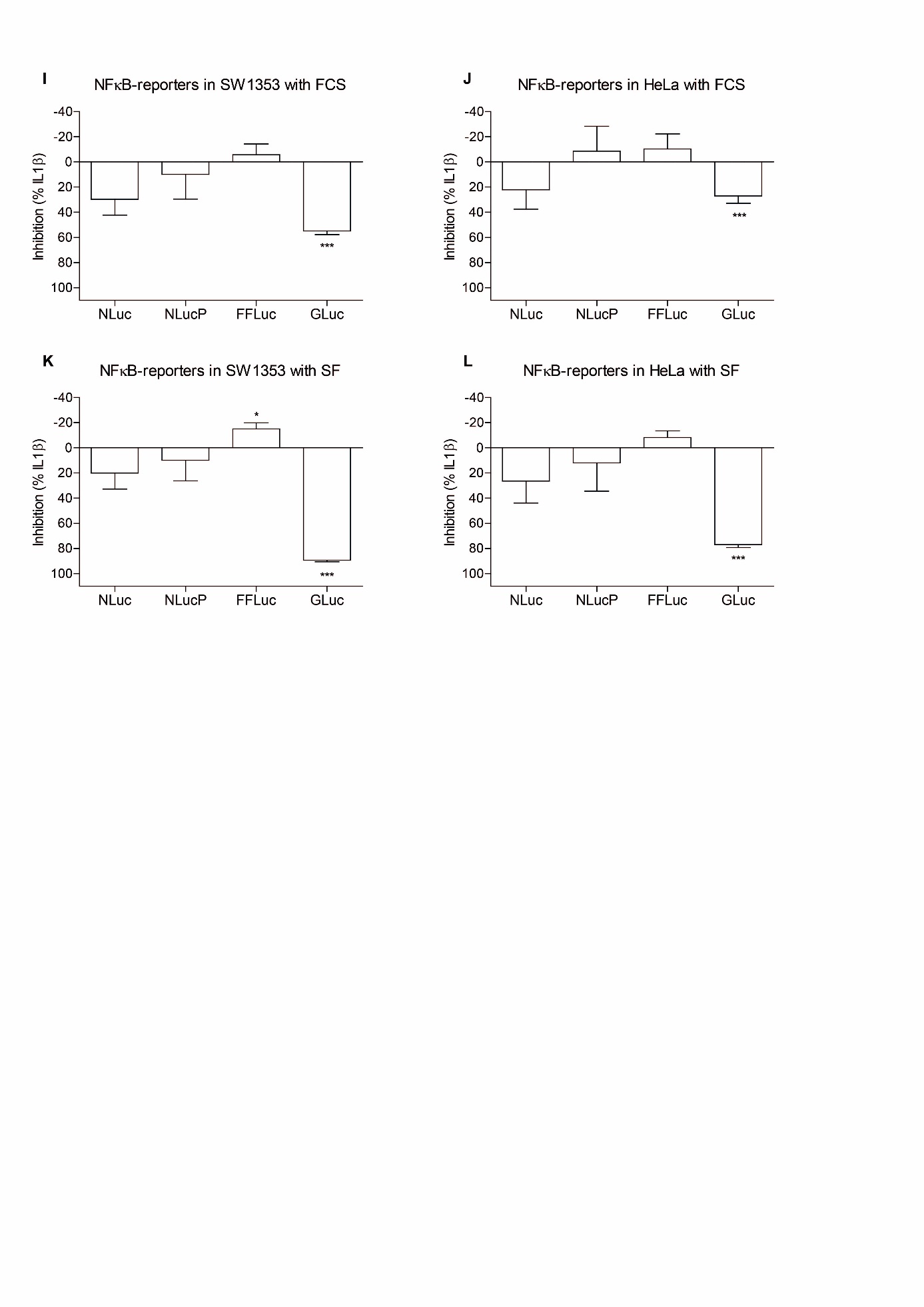


**Supplementary Figure 2**. **Complex body fluid interference with secreted Gaussia luciferase.** Fold change was determined at t_max_ of each specific reporter system. NFκB-RE reporters were stimulated with different concentrations of FCS (5%,10% or 20%) in (A) SW1353 cells and (B) HeLa cells and with different concentrations of SF (5%,10% or 20%) in (C) SW1353 cells and (D) HeLa cells. NFκB-RE reporters were stimulated with IL1β (1 ng/mL) with or without supplementation of 10% body fluid. Fold change of (E) NFκB-reporters in SW1353 cells with FCS, (F) NFκB-reporters in HeLa cells with FCS, (G) NFκB-reporters in SW1353 cells with SF and (H) NFκB-reporters in HeLa cells with SF. Percentage inhibition of stimulation with IL1β in combination with body fluid compared to IL1 β stimulation alone was determined in (I) SW1353 cells with FCS, (J) HeLa cells with FCS, (K) SW1353 cells with SF and (L) HeLa cells with SF. FCS; Fetal Calf Serum, SF; Synovial Fluid Data represents mean ± SD of four biological replicates. ** p. value ≤0.01,*** p. value ≤0.001.
