# Supplementary material for: Reporter gene comparison demonstrates interference of complex body fluids with secreted luciferase activity
Source: Sci Rep. 2021 Jan 14;11:1359. doi: 10.1038/s41598-020-80451-6 (PMC7809208; doi:10.1038/s41598-020-80451-6)
Supplement: Supplementary file 3 — Supplementary Information 3. [file 41598_2020_80451_MOESM3_ESM.docx]

**
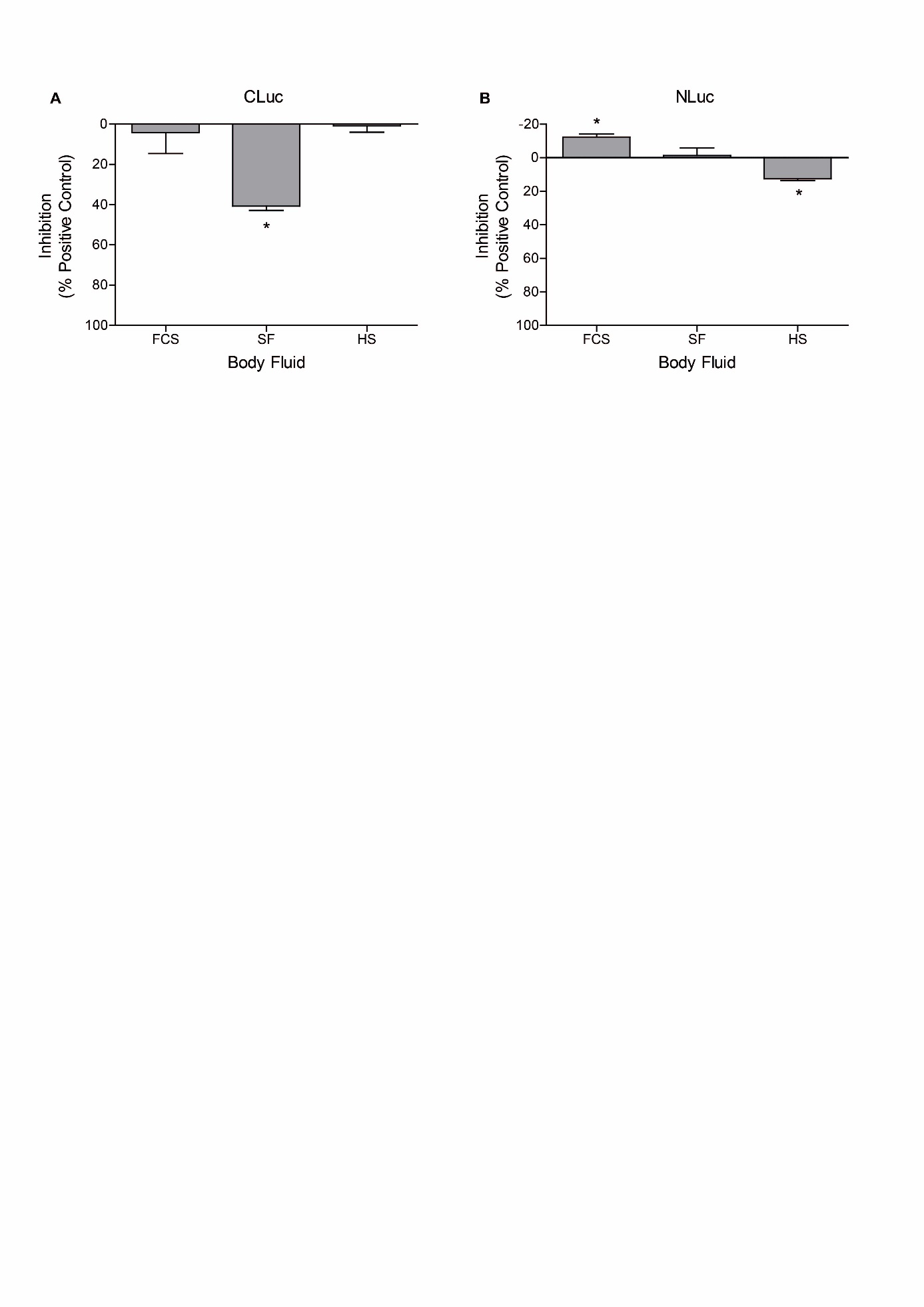
**

**Supplementary Figure 3. Inhibition of other secreted luciferases with complex body fluids.** Different kinds of body fluids (HS, FCS or SF) were added to a (A) CLuc containing medium sample to a final concentration of 10% 0r to a (B) NLuc containing medium sample to a final concentration of 10% .
