# Supplementary material for: Reporter gene comparison demonstrates interference of complex body fluids with secreted luciferase activity
Source: Sci Rep. 2021 Jan 14;11:1359. doi: 10.1038/s41598-020-80451-6 (PMC7809208; doi:10.1038/s41598-020-80451-6)
Supplement: Supplementary file 4 — Supplementary Information 4. [file 41598_2020_80451_MOESM4_ESM.docx]

**SUPPLEMENTARY FILES TO**

**Reporter gene comparison demonstrates interference of complex body fluids with secreted luciferase activity**

**M. Neefjes^1†^, B.A.C. Housmans^2†^, G.G.H. van den Akker^2^, L.W. van Rhijn^3^, T.J.M. Welting^2,3*†^, P.M. van der Kraan^1†^**

^1^Experimental Rheumatology, Department of Rheumatology, Radboud University Medical Centre

^2^Laboratory for Experimental Orthopedics, Department of Orthopedic Surgery, Maastricht University

^3^ Laboratory for Experimental Orthopedics, Department of Orthopedic Surgery, Maastricht University Medical Centre+

† These authors contributed equally

^*^Corresponding author

| **Significance table between Donors - Fetal Calf Serum** | | | | | |  |  |
| --- | --- | --- | --- | --- | --- | --- | --- |
| Donor | 1 | 2 | 3 | 4 | 5 | 6 | 7 |
| 1 |  | ns | ns | ns | ns | ns | ns |
| 2 | ns |  | ns | * | ns | ns | ns |
| 3 | ns | ns |  | ns | ns | ns | ns |
| 4 | ns | * | ns |  | ns | ns | * |
| 5 | ns | ns | ns | ns |  | ns | ns |
| 6 | ns | ns | ns | ns | ns |  | ns |
| 7 | ns | ns | ns | * | ns | ns |  |
|  |  |  |  |  |  |  |  |
| **Significance table between Donors - Synovial Fluid** | | | | |  |  |  |
| Donor | 1 | 2 | 3 | 4 | 5 | 6 | 7 |
| 1 |  | ns | ns | ns | ns | *** | ns |
| 2 | ns |  | ns | ns | ns | ** | ns |
| 3 | ns | ns |  | ns | ns | *** | ns |
| 4 | ns | ns | ns |  | ns | ** | ns |
| 5 | ns | ns | ns | ns |  | *** | ns |
| 6 | *** | ** | *** | ** | *** |  | *** |
| 7 | ns | ns | ns | ns | ns | *** |  |
|  |  |  |  |  |  |  |  |
| **Significance table between Donors - Human Serum** | | | | |  |  |  |
| Donor | 1 | 2 | 3 | 4 | 5 | 6 | 7 |
| 1 |  | ns | *** | *** | ns | ** | ns |
| 2 | ns |  | *** | *** | ns | *** | * |
| 3 | *** | *** |  | *** | *** | *** | *** |
| 4 | *** | *** | *** |  | *** | ns | ** |
| 5 | ns | ns | *** | *** |  | *** | *** |
| 6 | ** | *** | *** | ns | *** |  | ns |
| 7 | ns | * | *** | ** | *** | ns |  |

**Supplemental Table 1: Inter-donor statistics figure 4**

Inter-donor significance for the different kinds of body fluids. *p≤0.05 **, p≤0.01,*** p ≤0.001, ns; not significant.
